# *Cirsiumtatakaense* (Compositae), a new species from Taiwan

**DOI:** 10.3897/phytokeys.117.29380

**Published:** 2019-02-14

**Authors:** Chih-Yi Chang, Hsy-Yu Tzeng, Yen-Hsueh Tseng

**Affiliations:** 1 Department of Forestry, National Chung-Hsing University, No. 145, Hsing-Ta Rd., Taichung 402, Taiwan National Chung-Hsing University Taichung Taiwan

**Keywords:** New species, Chromosome number, *
Cirsium tatakaense
*, *
Cirsium kawakamii
*, Compositae, Taiwan

## Abstract

A new species of *Cirsium*, *Cirsiumtatakaense* Y.H.Tseng & C.Y.Chang, from central-southern Taiwan is described and illustrated. This species is similar to *C.kawakamii* Hayata in leaf shape, achene and chromosome number (2n = 64), but can be readily distinguished from *C.kawakamii* by the narrower leaf lobes, usually higher number of florets and phyllaries, the purplish-red corolla (vs. white) and larger pollen grains. A key to the species of *Cirsium* in Taiwan is also presented.

## Introduction

*Cirsium* Mill. is a genus of Compositae and comprises approximately 250 species widely distributed throughout the world’s temperate and subtropical zones (Kadota 2007). A total of 46 species are native to China (Shih and Greuter 2011), 64 to Japan (Iwatsuki et al. 1995) and 9 to Taiwan (Peng 2003).

Kitamura (1937) established the first infrageneric classification for East Asian *Cirsium*. In this classification, the Taiwanese species were placed in two of the sections, sect. Pseudoeriolepis (Nakai) Kitam. and sect. Onotrophe (Cass.) DC. and five subsections, subsect. ArenicolaKitam.,subsect.Australicirsium Kitam., subsect. NipponocirsiumKitam.,subsect.Sinocirsium Kitam. and subsect. Spanioptilon Less. The latter subsection was subsequently raised to the rank of section (sect.Spanioptilon (Less.) Shih) by Shih (1984) and maintained by Iwatsuki et al. (1995).

Recently, we discovered a rare *Cirsium* growing in the high mountain areas of central-southern Taiwan. Based on the systems of Kitamura (1937) and Iwatsuki et al. (1995), this taxon belonged to section OnotrophesubsectionNipponocirsium and appeared to be similar to *Cirsiumkawakamii* Hayata, sharing the longer leaf lobes, nodding bowl-shaped mature capitula and corolla lobes as long as the inflated part of the corolla tube.

## Materials and methods

We compared the new species to the other two species of Cirsiumsect.Onotrophe in Taiwan.

### Herbarium examination

Materials primarily comprised fresh and dried specimens; voucher specimens were deposited in TCF, PPI and TNM. The herbaria referenced included HAST, KYO, PPI, TAI, TAIF, TCF, TI and TNM.

### Pollen morphology

Pollen grains, all from fresh material, were directly mounted on a stub without any pretreatment and sputter coated with gold (Quorum SC7620) for observation with a scanning electron microscope (Hitachi S-3400N). The shape, size and exine ornamentation were studied following Erdtman (1952) and Hesse et al. (2009). Vouchers for the pollen material studied are provided in Table 1.

**Table 1.** Voucher material for the *Cirsium* Mill. pollen morphology.

Taxa	Location	Coordinate	Altitude	Date	Voucher
* C. tatakaense *	TAIWAN. Kaohsiung City Tauyuan District, Kuaiku to Yakou	23°16.03'N, 120°56.24'E	2600 m	21 Oct 2014	*C. Y. Chang*, 160 (TNM)
	TAIWAN. Nantou County Sinyi Township, Tataka	23°29.24'N, 120°52.71'E	2620 m	15 Nov 2014	*C. Y. Chang 182* (TCF)
	TAIWAN. Nantou County Sinyi Township, Tataka to Shihshan	23°28.56'N, 120°52.74'E	2530 m	26 Oct 2015	*C. Y. Chang*, *842* (TNM)
	TAIWAN. Nantou County Sinyi Township, Tataka to Shihshan	23°29.24'N, 120°52.71'E	2631 m	22 Dec 2015	*C. Y. Chang 1017* (TCF)
* C. kawakamii *	TAIWAN. Taichung City Heping District, Mt. Pintian to Mt. Dabajian	24°26.52'N, 121°15.83'E	2800 m	13 Aug 2014	*C. Y. Chang 264* (TNM)
	TAIWAN. Taichung City Heping District, Mt. Syue Trail 8.9 km	24°23.53'N, 121°14.20'E	3500 m	3 Oct 2015	*C. Y. Chang 774* (TCF)
	TAIWAN. Nantou County Ren’ai Township. Nengao cross-ridge historic trail 11 km	24°2.79'N, 121°15.77'E	2600 m	29 Jan 2018	*C. Y. Chang 1605* (TNM)
* C. arisanense *	TAIWAN. Taichung City Heping District, Mt. Syue trail 8.9 km	24°23.60'N, 121°13.98'E	3450 m	3 Sept 2015	*C. Y. Chang 756* (TCF)
	TAIWAN. Nantou County Ren’ai Township, Rueiyan river pipes road 2 km	24°06.95'N, 121°11.96'E	2240 m	27 May 2016	*C. Y. Chang 1275* (TCF)

### Karyotype analysis

Karyotype analysis was performed by following the same procedure applied by Ozcan et al. (2011) and Yüksel et al. (2013). Root tips were collected on sunny mornings and preserved in 0.002 M 8-hydroxyquinoline solution below 10 °C for eight hours. This material was then fixed with Carnoy’s solution (1 part acetic acid: 3 parts EtOH) for at least 24 hours at 4 °C. The fixed roots were then stained with acetic-orcein for 24 hours at room temperature, squashed and the slides examined using an stereo microscope (ACCU-ScoPE 3025). Voucher material is presented in Table 2.

**Table 2.** Voucher material for the *Cirsium* Mill. karyotype analysis.

Taxa	Location	Coordinate	Altitude	Date	Voucher
* C. tatakaense *	TAIWAN. Chiayi County Alishan Township, Tataka to Paiyun lodge	24°28.40'N, 120°54.23'E	2800 m	15 May 2016	*C. Y. Chang 1269* (TCF)
* C. kawakamii *	TAIWAN. Taichung city Heping District, Mt. Syue trail 8.9 km	24°23.73'N, 121°13.94'E	3371 m	8 Nov 2015	*C. Y. Chang 874* (TCF)
	TAIWAN. Taichung city Heping District, Mt. Syue trail 8.9 km	24°23.73'N, 121°13.94'E	3371 m	21 May 2016	*C. Y. Chang 1271* (TCF)

## Results and discussion

### Morphological comparison

Following Kitamura (1937) and Iwatsuki et al. (1995), *Cirsiumtatakaense* is placed in sectionOnotrophe together with *C.kawakamii* and *C.arisanense.* Amongst them, *C.arisanense* belongs to subsection Australicirsium, which is characterised by having rosette leaves, pot-shaped capitula which are erect or nodding when mature, corolla lobes equal in length to the inflated part of corolla tube and corona-like achene beaks. Both *C.tatakaense* and *C.kawakamii* belong to subsection Nipponocirsium, which is characterized by not having rosette leaves, larger bowl-shaped capitula which are nodding when mature, corolla lobes equal in length to the inflated corolla tube and tube-like achene beaks. In comparison with *C.kawakamii* (Table 3), *C.tatakaense* has purplish corolla (vs. white in *C.kawakamii*) (Figure 5[2]), usually more florets (136)161−308 (vs. (61)115−222 in *C.kawakamii*) (Figure 5[2]) and phyllaries 111−199 (vs. 79−123 in *C.kawakamii*) (Figure 5[2]) and narrower leaf lobes 7.3−11.9 mm (vs. 17.2−18.6 mm in *C.kawakamii*) (Figure 5[1]).

**Table 3.** Summary of characters between the species of Cirsiumsect.Onotrophe in Taiwan.

Characters	* C. tatakaense *	* C. kawakamii *	* C. arisanense *
Leaf size (cm)	27.2−34.8 cm × 16.4−19.4 cm	27.5−30.6 cm × 17.8−20.2 cm	10.6−21.3 cm × 3.0−6.3 cm
Leaf shape	Elliptic to broadly elliptic	Elliptic to broadly elliptic	Narrowly elliptic to deltoid
Leaf margin	Mainly pinnatisect	Pinnatisect or bipinnatisect	Pinnatipartite or bipinnatipartite
Leaf lobes Size	6.4−7.5 cm × 7.3−11.9 mm	8.3−10.4 cm × 17.2−18.6 mm	0.7−2.9 cm × 6.6−16.0 mm
Pair of leaflobes	4−6	6−7	6−10
Mature Capitula	Nodding	Nodding	Erect or nodding
Involucre shape	Bowl-shaped (upper width ≥ base)	Bowl-shaped (upper width ≥ base)	Pot-shaped (upper width< base)
Corolla colour	Purplish-red	White	Yellow
Floret number	(136)161−308	(61)115−222	87−133
Phyllary number	111−199	79−123	93−114
Beak of achene	Tube-like	Tube-like	Corona-like
Pollen size (P/E)	34.2−42.6μm/ 35.2−44.7 μm	31.7−34.5 μm/ 34.3−37.1 μm	41.7−51.0 μm/ 44.7−49.3 μm
Pollen spine base width	4.2−5.6 μm	2.0−2.3 μm	2.8−4.8 μm
Chromosome number	2n = 64	2n = 64	2n = 34 (Peng and Hsu 1978)
Distribution	Endemic to Taiwan; open areas of fog forests at 2000−3000 m alt. central-southern Taiwan (Fig. 3)	Endemic to Taiwan; gullies and valleys at 1500−3500 m alt. central-northern Taiwan (Fig. 3)	Endemic to Taiwan; widely distributed in open areas of mountain area at 1500−3800 m alt. (Fig. 3)

### Chromosome number

The basic number of chromosomes amongst *Cirsium* species is often 2n = 34 (Hsu 1970; Funk et al. 2009; Chen and Yeh 2010a; Chen and Yeh 2010b), including in *C.arisanense* (Peng and Hsu 1978). However, the chromosome number of *C.tatakaense* is 2n = 64 (Fig. 6A), which is the same as that of *C.kawakamii* (Fig. 6B), indicating that the two species are similar in this respect. Notably, other taxa of the same subsection in Japan are 2n = 68 (Iwatsuki et al. 1995). These findings imply that subsect. Nipponocirsium are tetraploids with aneuploid cells.

### Palynological study

*Cirsiumtatakaense* pollen has a larger diameter, up to 36−43 μm (vs. 32−35 μm in *C.kawakamii*) and its surface spines have broader bases of 4.2−5.6 μm (vs. 2.0−2.3 μm in *C.kawakamii*). The pollen grains of *C.tatakaense* are similar to *C.arisanense* (Fig. 7A, C). However, *C.kawakamii* (similar to *C.tatakaense* in macroscopic morphology) has the smallest pollen grains and spine in Taiwan (Fig. 7B). Pollen morphology is associated with pollination, thus implying reproductive isolation between the two species.

### Comparison of the distribution between *C.tatakaense* and *C.kawakamii*

Compared with *C.tatakaense*, *C.kawakamii* occurs at higher altitudes (up to 3500 m); *C.tatakaense* is seldom discovered over altitudes of 3000 m. In addition, *C.kawakamii* is usually distributed in alpine gullies and valleys, whereas *C.tatakaense* often appears on spacious roadsides, seemingly with no preference for valley habitats. Therefore, we believe that *C.kawakamii* prefers shaded and moist environments, whereas *C.tatakaense* prefers open areas with higher drought tolerance. Some geographical segregation appears to exist in the distributions of *C.tatakaense* and *C.kawakamii*.

### Taxonomic treatment

#### Key to the species of *Cirsium* Mill. in Taiwan

**Table:** 

1	Biennial herb; involucre tube-shaped (length 2 times than width); corolla lobes < 2.5 mm long	*** C. ferum ***
–	Perennial herb; involucre pot or bowl-shaped (length approximates width), corolla lobes > 2.5 mm long	**2**
2	All leaves cauline, basal rosette leaves absent	**3**
–	Leaves in both a basal rosette as well as cauline	**5**
3	Leaves densely cobwebbed on abaxial surface; mature capitula erect, involucre pot-shaped (upper width shorter than base); apical parts of inner phyllaries inflated, obtuse; outer phyllaries lanceolate, apex acute without spine; corolla lobes obviously longer than the inflated part of corolla tube	*** C. lineare ***
–	Leaves glabrous on both surfaces; mature capitula nodding, involucre bowl-shaped (upper width greater or equal to base); apical parts of inner phyllaries acute or acuminate; outer phyllaries elliptic with long spine at the apex; corolla lobes as long as the inflated part of corolla tube	**4**
4	Corollas white; leaves pinnatisect or bipinnatisect, lobes > 15 mm wide	*** C. kawakamii ***
–	Corollas purple; leaves mainly pinnatisect, lobes < 12 mm wide	*** C. tatakaense ***
5	Phyllaries narrowly ovate	**6**
–	Phyllaries subulate	**7**
6	Corollas white; phyllaries lanceolate, inner and outer phyllaries similar in length; stems cauline, without rhizome	*** C. brevicaule ***
–	Corollas purple; phyllaries narrowly ovate to ovate, inner and outer phyllaries distinct in length; stems both cauline and rhizomatous	*** C. morii ***
7	Apical prominently parts of phyllaries longer than 4 mm, blade-like; corolla lobes as long as the inflated part of corolla tube	**8**
–	Apical prominently parts of phyllaries shorter than 4 mm, spine-like; corolla lobes shorter than the inflated part of corolla tube	**9**
8	Leaf abaxial surface pubescent; mature capitula erect or nodding	*** C. arisanense ***
–	Leaf abaxial surface densely cobwebbed; mature capitula nodding	*** C. hosokawae ***
9	Leaf abaxial surface densely cobwebbed; mature capitula nodding	*** C. suzukii ***
–	Leaves surface pubescent; mature capitula erect	**10**
10	Corollas purple; leaves surface shortly hairy	** C. japonicum var. australe **
–	Corollas white; leaves surface glabrescent	** C. japonicum var. takaoense **

#### Species treatments

##### Cirsium kawakamii

Taxon classificationPlantaeAsteralesAsteraceae

Hayata in J. Coll. Sci. Imp. Univ. Tokyo. 159. 1911.

Figs 4B , 5B , 6B , 7B

###### Type.

TAIWAN. Mt. Morrison, ca. 3000 m alt., 20 Oct.1906. *T. Kawakami & U. Mori 2279* (holotype: TI!; isotype: TAIF!).

###### Description.

Perennial herbs, stems 0.5−1.8 m tall, without rosette leaves. Leaves pinnatipartite or pinnatisect, 27.5−30.6 cm long and 17.8−20.2 cm wide, U-shaped space between pinnae, smooth, elliptic to broadly elliptic, base truncate to cuneate, apex caudate, pinnae 8.3−10.4 cm long and 17.2−18.6 mm wide, space between pinnae 2.5−3.0 cm, 6−7 pairs. Capitula arranged into racemes or panicles, mature capitula nodding, involucre bowl shaped, 3.4−3.8 cm long and 1.5−2.0 cm wide. Involucre lacking abaxial appendages, inner phyllaries acute apically, outer phyllaries green with indistinct layers, 1.6−1.8 cm long and 1.8−2.4 mm wide, protrusion 6.0−11.0 mm. Florets with white corolla, 2.8−3.1 cm long, corolla lobes 5.2−6.0 mm long and 0.4−0.7 mm wide; 5 synantherous stamens, detached filaments with irregular protuberances, basal caudate extensions, white or brown, anthers 5.4−8.2 mm long, filaments 6.8−8.0 mm long. Stigmas bifid apically, style 2.0−3.4 cm long, ovaries 1.5−2.0 mm long. Achenes oblong, base acute, apex truncate, beige, 4.3−4.9 mm long and 1.7−1.8 mm wide, long tube-shaped beak apically. Pappus 1.3−2.1 cm long forming basal ring, easily shed.

###### Phenology.

Flowering between September and October and fruiting between October and November.

###### Distribution.

Endemic to central-northern Taiwan. Preference for gullies and valleys at 1500−3500 m alt. (Fig. 3).

###### Chinese name.

Yu-shan-ji (玉山薊).

###### Chromosome number.

2n=64 (Fig. 6B).

###### Palynology.

Pollen grains are tricolporate, spheroidal, microreticulate and 31.7−34.5 × 34.3−37.1 μm (P/E ratio: 0.9−1.0). The surface is densely covered with spines that are 2.5−3.2 μm long and 2.1−2.2 μm wide at the base. The distance between spines is 7.6−8.8 μm (Fig. 7B).

###### Additional specimen examined.

TAIWAN. Taoyuan City, Fuxing District, Mt. Lalashan, 1550−1700 m alt., 25 Sept. 1991. *C. I Peng 14628* (HAST!). Miaoli County, Tai’an Township, Tunnel of Mt. Shishihshan to Mt. Huoshihshan, 2480 m alt., 18 Sept. 1995. *C. M. Wang 1728* (TNM!). Taichung City, Heping District, Mt. Syue to Mt. Chihchiayangdashan, 3300 m alt., 10 Sept. 2014. *C. Y. Chang et C. H. Liu 68* (TNM); Mt. Pintian to Mt. Dabajianshan, 2800 m alt., 24°26.52'N, 121°15.83'E, 13 Aug. 2014. *C. Y. Chang 264* (TNM); Mt. Syue Trail 8.9 km, 3500 m alt., 24°23.53'N, 121°14.20'E, 3 Oct 2015. *C. Y. Chang 774, 1271* (TCF). Nantou County, Ren’ai Township, Nengao cross-ridge historic trail 11 km, 2600 m alt., 24°2.79'N, 121°15.77'E, 29 Jan 2018. *C. Y. Chang 1605* (TNM); Chengkung lodge, 3140 m alt., 31 July 2015. *C. Y. Chang 654* (TNM); Guandao river, 22 Oct. 1932. *S. Sasao s. n.* (CHIA!); Mt. Hohwanshan, 3300 m alt., 15 Oct. 1994. *Tunghai Collecting Team s. n.* (TNM!). Hualien County, Xiulin Township, Sungshiuelou lodge to Dayuling, 3 Aug. 1974. *C. N. Lin s. n.* (KYO!).

##### Cirsium tatakaense

Taxon classificationPlantaeAsteralesAsteraceae

Y.H.Tseng & C.Y.Chang sp. nov.

urn:lsid:ipni.org:names:77194985-1

**Figure 1.**
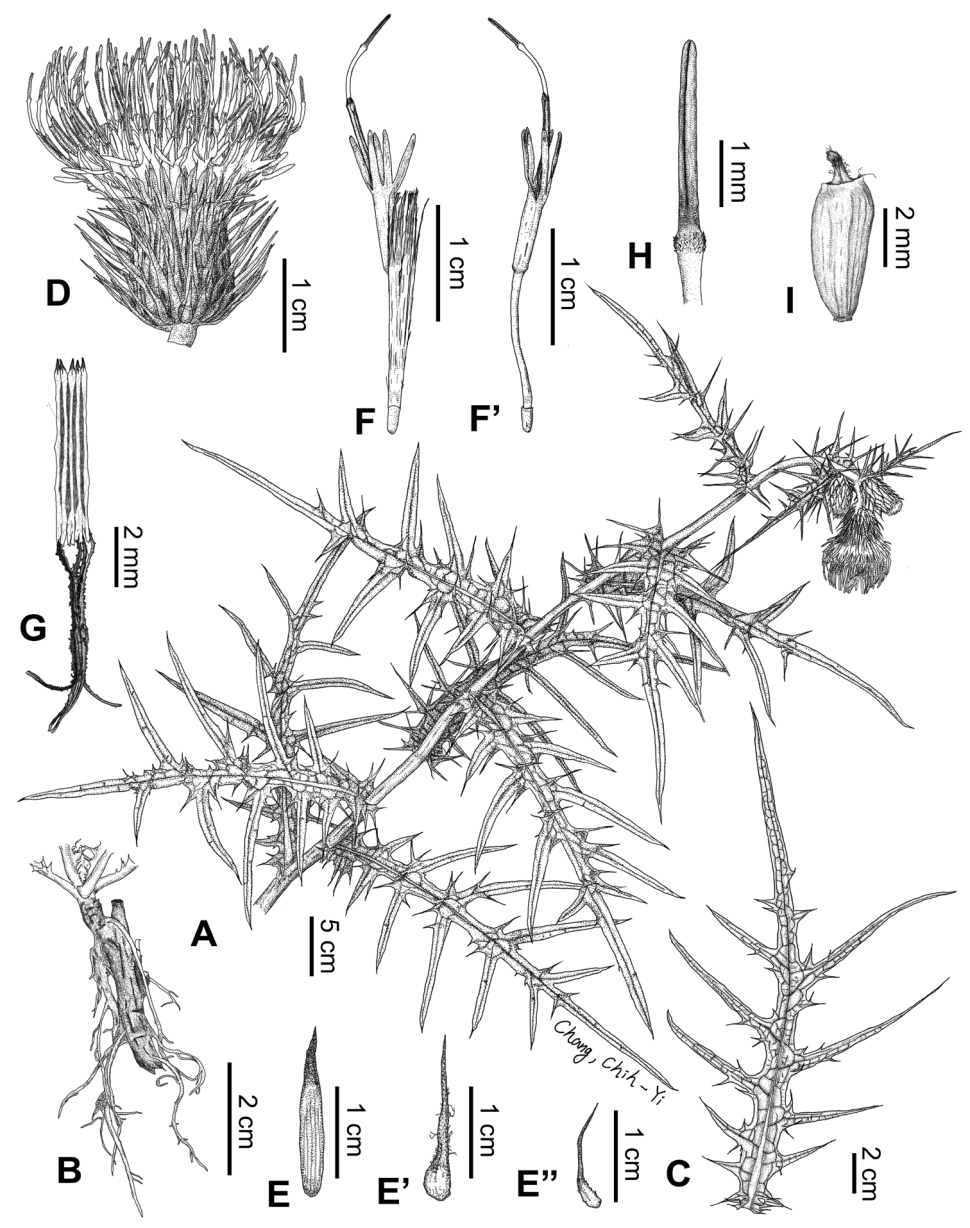
Line drawings of *Cirsiumtatakaense* Y.H.Tseng & C.Y.Chang **A** habit **B** root **C** leaf **D** capitula **E** inner phyllary **E**’ middle phyllary **E**” outer phyllary **F** floret **F**’ floret (pappus removed) **G** synantherous **H** style branches **I** achene. Voucher: *C. Y. Chang 1442* (TCF).

**Figure 2.**
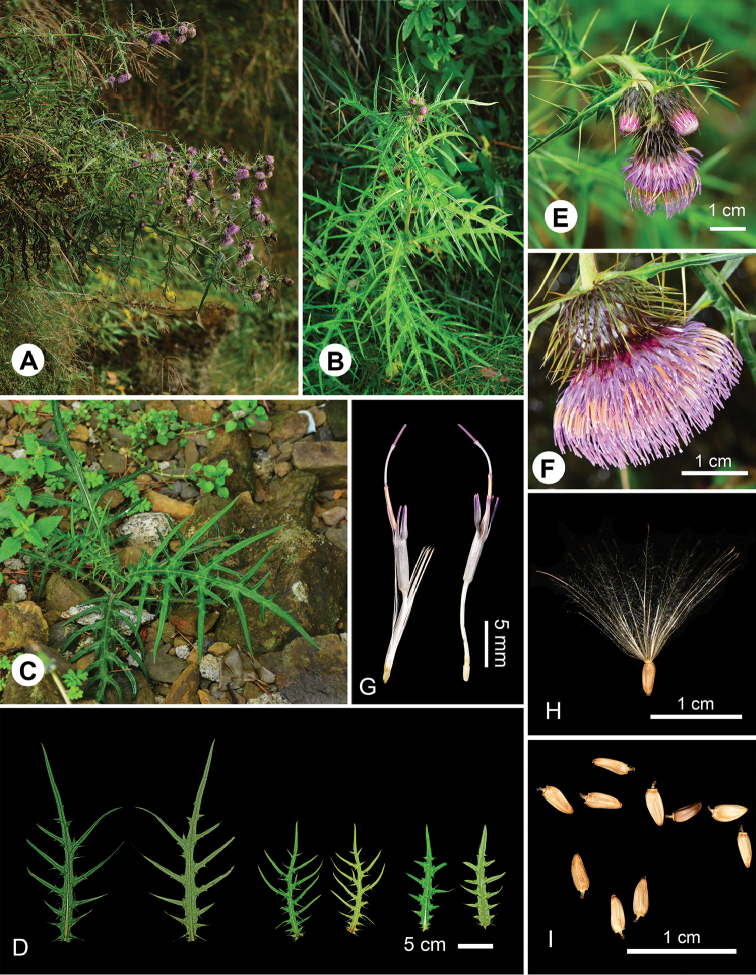
*Cirsiumtatakaense* Y.H.Tseng & C.Y.Chang **A** habitat **B** habit **C** seedling **D** variations of leaves **E** inflorescences **F** capitula **G** floret; (right-side pappus removed) **H** achene with pappus **I** achene.

Figs 1 , 2 , 4A , 5A , 6A , 7A

###### Diagnosis.

Differs from *C.kawakamii* in having narrower leaf lobes (7.3−11.7 mm), usually more florets, (136)161−308 and phyllaries (111−199), a purplish-red corolla and larger pollen grains (34.2−42.6 × 35.2−44.7 μm).

###### Type.

TAIWAN. Nantou County, Sinyi Township, Highway no. 18, Tataka to Shihshan, 2400 m alt., 23°28.52'N, 120°52.10'E, 3 October 2016. *C. Y. Chang 1444* (holotype: TCF; isotype: TNM, PPI).

###### Description.

Perennial herbs, stems 0.5−1.5 m tall, without rosette leaves. Leaves pinnatipartite or pinnatisect, 27.2−34.8 cm long and 16.4−19.4 cm wide, U-shaped space between pinnae, smooth, elliptic to broadly elliptic, base truncate to cuneate, apex caudate, pinnae 6.4−7.5 cm long and 7.3−11.9 mm wide, space between pinnae 2.9−3.0 cm, 4−6 pairs. Capitula arranged into racemes or panicles, mature capitula nodding, involucre bowl-shaped, 3.9−4.0 cm long and 1.7−2.1 cm wide. Involucre lacking abaxial appendages, inner phyllaries acute apically, outer phyllaries reddish-purple with indistinct layers, 1.1−2.2 cm long and 1.8−2.4 mm wide, protrusion 6.3−13.0 mm. Florets with purplish-red corolla, 3.2−3.3 cm long, corolla lobes 4.3−5.3 mm long and 0.4−0.7 mm wide; 5 synantherous stamens, detached filaments with irregular protuberances, basal caudate extensions, light purple or brown, anthers 6.4−6.8 mm long, filaments 7.1−8.1 mm long. Stigmas bifid apically, style 3.0−3.2 cm long, ovaries 1.7−1.9 mm long. Achenes oblong, base acute, apex truncate, beige, 3.0−3.5 mm long and 1.4−1.6 mm wide, long tube-shaped beak apically. Pappus 1.6−1.8 cm long forming basal ring, easily shed.

###### Phenology.

Flowering between August and October and fruiting between September and November.

###### Distribution.

**Figure 3.**
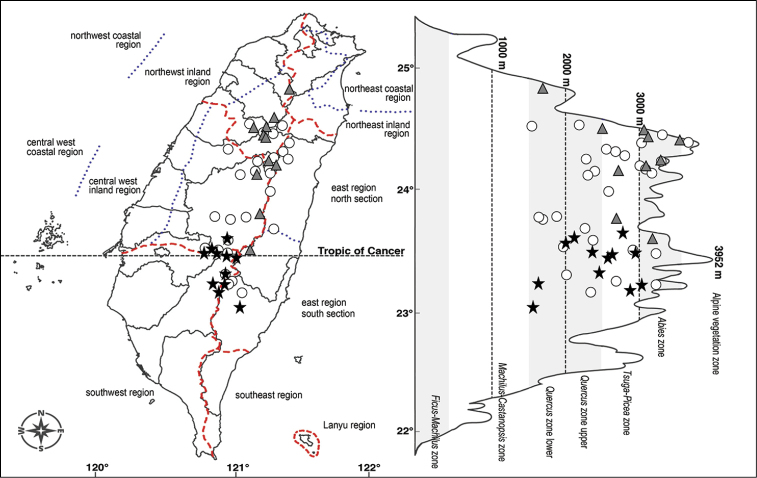
Distribution map of *Cirsiumtatakaense* Y.H.Tseng & C.Y.Chang (star); *C.kawakamii* Hayata (triangle); and *C.arisanense* Kitam. (circle) of Taiwan.

Endemic species of Taiwan. *Cirsiumtatakaense* is located in open areas of cloud forests of vegetation zones from the *Quercus* to *Abies* forest zone at alt. 2000−3000 m in central-southern Taiwan (Fig. 3). Based on the geographical climatic regions and vegetation zones (Su 1984, 1985), *C.tatakaense* is distributed mainly in the central-west inland regions. *Cirsiumtatakaense* has been discovered in sunny environments, such as roadsides and forest margins, concentrated on the upper portions of hills along Provincial Highway no. 18. *Miscanthustransmorrisonensis* Andersson (Poaceae), *Rubustaitoensis* Hayata (Rosaceae) and SenecionemorensisL.var.dentatus (Kitam.) H. Koyama (Compositae) are often discovered with *C.tatakaense*. Sometimes, *C.arisanense* Kitam. and *C.ferum* Kitam. are found near to *C.tatakaense*; however, no hybrid individual between these species has been observed.

###### Chinese name.

Ta-ta-jia-ji (塔塔加薊).

###### Etymology.

The species epithet *tatakaense* derives from the type location Tataka in Nantou County.

###### Notes.

This species has in the past been mistakenly identified as *C.kawakamii* (*S. Saito 3477*, KYO!; *Yamazaki 945*, KYO!; *C. I Peng 8026, 8936, 11788, 14628*, HAST!; *K. F. Chung 1053*, HAST!; *Kawakami & Sasaki s. n.*, TAIF!; *M. L. Weng 1723*, TAI!; *Y. Kudo & S. Suzuki 300*, TAI!; *C. C. Hsu 4231*, TAI!; *C. I Peng 738*, TAI!; *C. T. Chao et al. 2534*, TNM!; *C. S. Kuoh 15146*, TNM!) or less often as *C.arisanense* Kitam. (*Yamamoto et al. 4142*, TAI!; *Y. J. Lin 169*, PPI!). The earliest record of *C.tatakaense* was collected by *T. Kawakami & S. Sasaki s. n.* (TAIF!) at Mt. Morrison (alt. ca. 3000 m) on October 8, 1909. Other specimens collected from 1909−1930 were from Alishan (alt. ca. 2200 m). However, only a few populations remain in Alishan, with the largest population appearing along the Yushan Main Peak Trail from Tataka to Paiyun Lodge (alt. ca. 2800 m). We assume that use in herbal medicine as well as climate change have reduced the population of *C.tatakaense*.

###### Chromosome number.

2n = 64 (Fig. 6A)

###### Palynology.

Pollen grains are tricolporate, spheroidal, microreticulate and 34.2−42.6 × 35.2−44.7 μm (P/E ratio: 0.9−1.0). The surface of the pollen is densely covered with spines that are 3.2−5.1 μm long and 4.2−5.6 μm wide at the base. The distance between spines is 7.5−10.6 μm (Fig. 7A).

###### Conservation status.

*Cirsiumtatakaense* is distributed in central-southern Taiwan, with a population of more than 1000 mature individuals. Its habitats are mainly located in high and sunny mountain areas and many of them are difficult to locate. Therefore, following the International Union for Conservation of Nature (IUCN) Categories and Criteria (IUCN 2014), we regard this species as Least Concern. However, long-term monitoring of its population is still required.

###### Additional specimens examined (paratype).

TAIWAN. Nantou County, Sinyi Township, Highway no. 18, Tataka to Shihshan, 2400 m alt., 23°28.52'N, 120°52.10'E, 3 Oct. 2016. *C. Y. Chang 1442* (TCF); same loc., 3 Oct. 2016. *C. Y. Chang 1443* (TCF); same loc., 12 Sept. 2012. *C. T. Chao et al. 2534* (TNM!); Tunpu Hot Spring to Kuankao, 1300−2600 m alt., 3 July 1985. *C. I Peng 8026* (HAST!). Chiayi County, Alishan Township, Alishan, 25 Dec. 1928. *Y. Kudo & S. Suzuki 300* (TAI!); Tatachia saddle to Paiyunshanchuang, 2700−3000 m alt., 9 Nov. 1985. *C. I Peng 8931* (HAST!); Tatachia saddle to Mt. Yushanchienfu, 2700−3100 m alt., 11 Nov. 1990. *P. J. Wu et al. s. n.* (TNM!). Kaohsiung City, Tauyuan District, Kuaiku to Yakou, 2600 m alt., 23°16.03'N, 120°56.24'E, 21 Oct. 2014. *C. Y. Chang 160* (TNM); Gingzin bridge, 14 Sept. 1999. *C. S. Kuoh 15146* (TAN!). Taitung County, Yanping Township, Yenping forest-road, 1500−1800 m alt., 2 July 2006. *Y. J. Lin 169* (PPI!).

**Figure 4.**
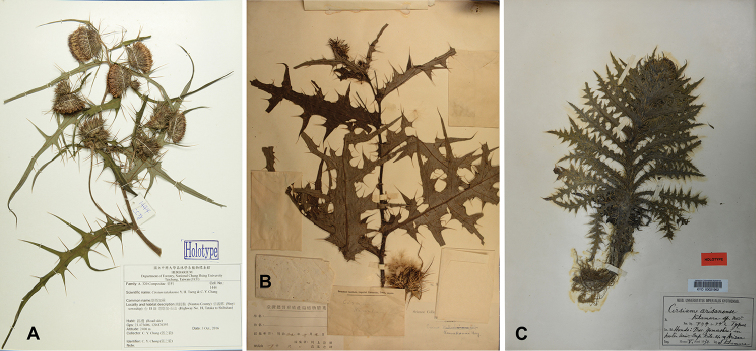
Holotypes of the three species of Cirsiumsect.Onotrophe in Taiwan. **A***C.tatakaense* Y.H.Tseng & C.Y.Chang, *C. Y. Chang 1444* (TCF) **B***C.kawakamii* Hayata, *T. Kawakami & U. Mori 2279* (TI!) **C***C.arisanense* Kitam., *S. Kitamura s.n.* (KYO!).

**Figure 5.**
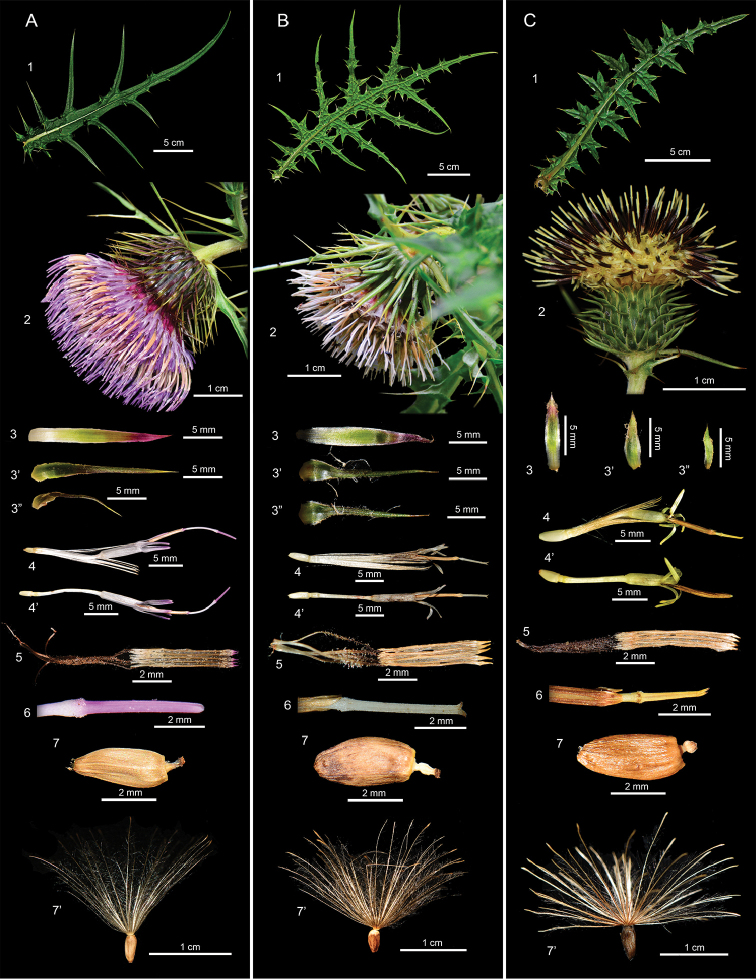
Comparison of the morphological characters amongst the species of Cirsiumsect.Onotrophe in Taiwan. **A***C.tatakaense* Y.H.Tseng & C.Y.Chang **B***C.kawakamii* Hayata **C***C.arisanense* Kitam.: **1** leaf **2** capitula **3** inner phyllary **3**’ middle phyllary **3**” outer phyllary **4** floret **4**’ floret (pappus removed) **5** synantherous **6** style branches **7** achene **7**’ achene with pappus.

**Figure 6.**
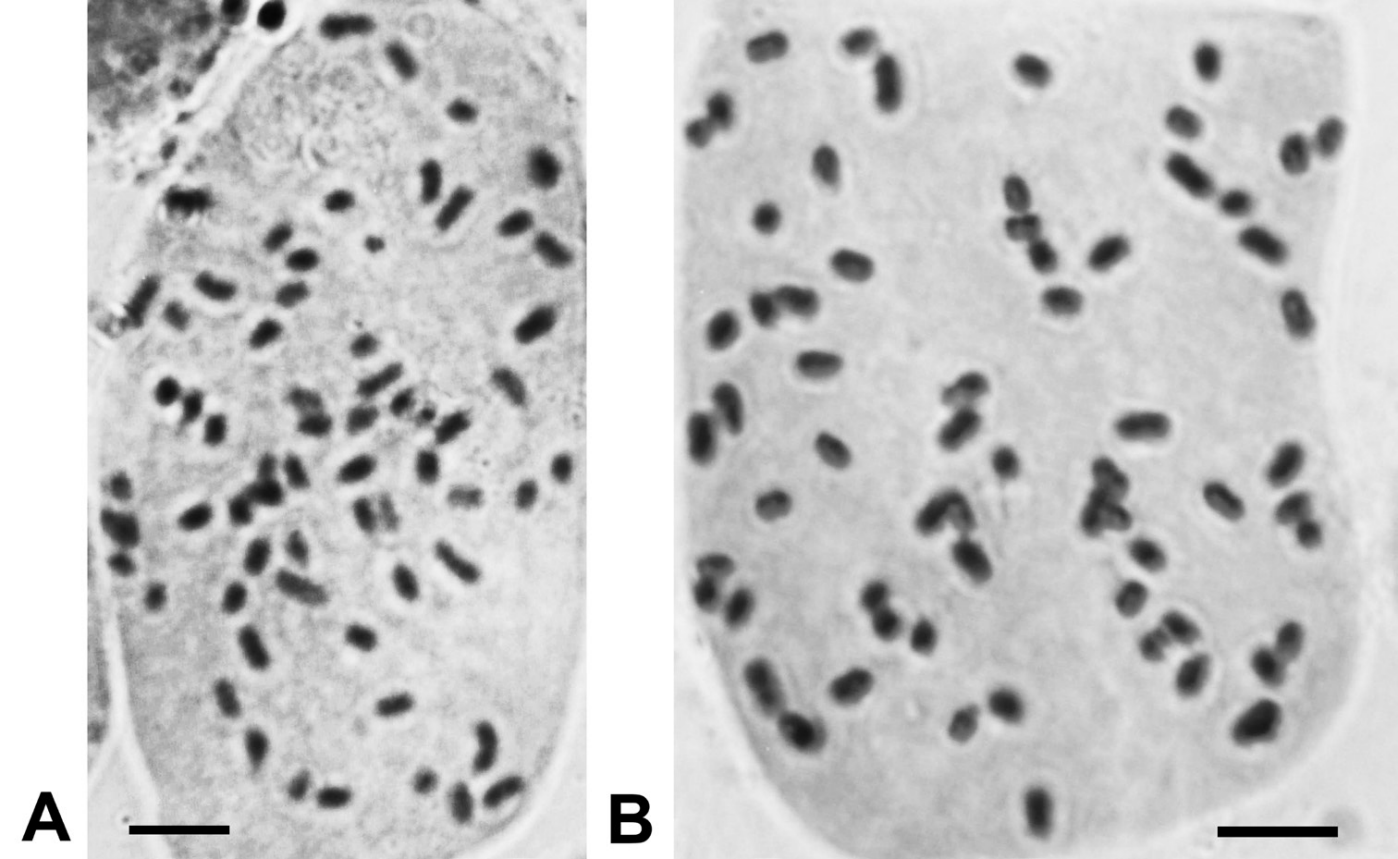
Chromosome number of the two species of Cirsiumsubsect.Nipponocirsium Kitam. in Taiwan. **A** 2n = 64, *C.tatakaense* Y.H.Tseng & C.Y.Chang **B** 2n = 64, *C.kawakamii* Hayata. Scale bar: 5 μm.

**Figure 7.**
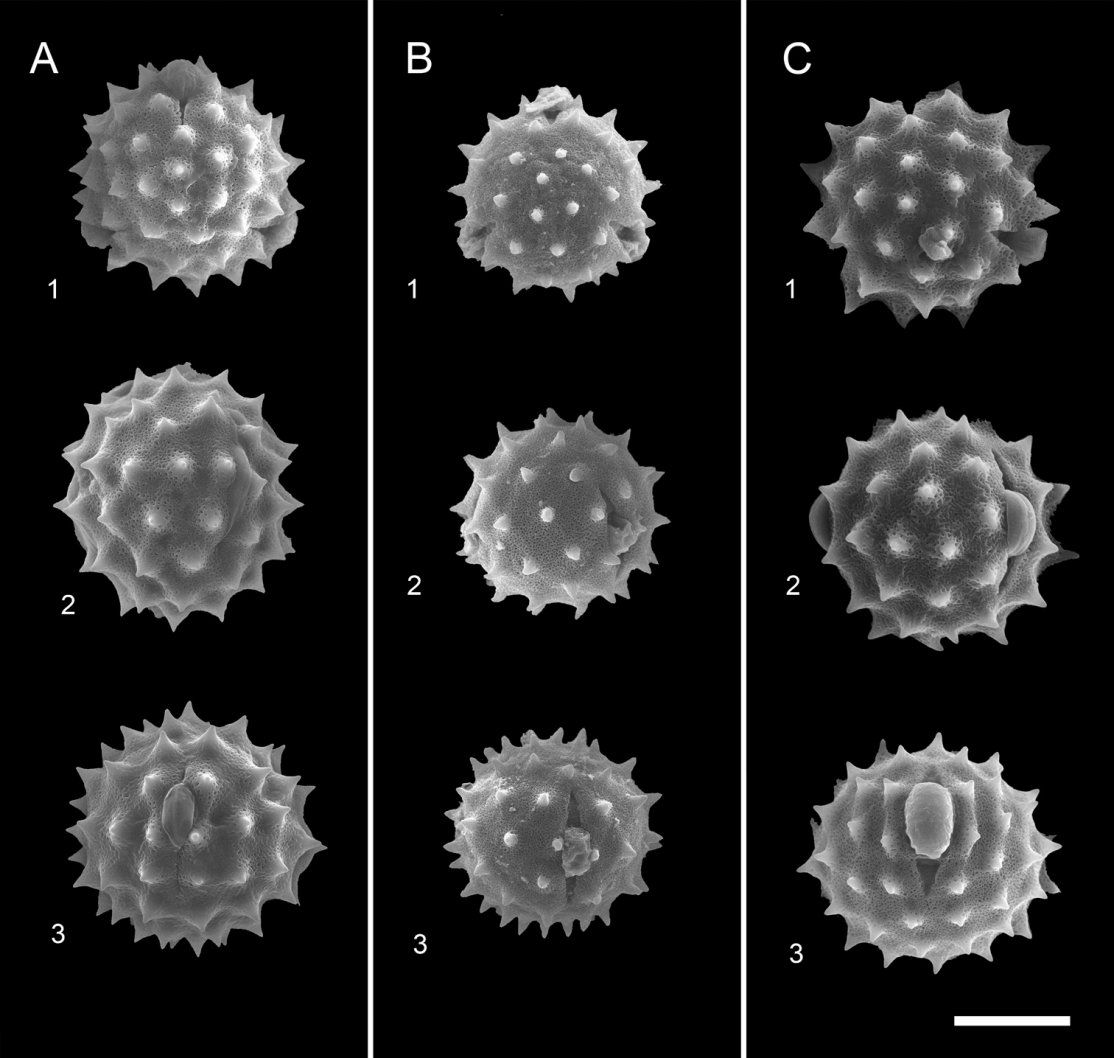
Comparison of the pollen morphology of the three species of Cirsiumsect.Onotrophe in Taiwan. **A***C.tatakaense* Y.H.Tseng & C.Y.Chang **B***C.kawakamii* Hayata **C***C.arisanense* Kitam.: **1** polar view **2** equatorial view **3** colporate view. Scale bar: 30 μm.

## Supplementary Material

XML Treatment for Cirsium kawakamii

XML Treatment for Cirsium tatakaense
